# No Benefit from Chronic Lithium Dosing in a Sibling-Matched, Gender Balanced, Investigator-Blinded Trial Using a Standard Mouse Model of Familial ALS

**DOI:** 10.1371/journal.pone.0006489

**Published:** 2009-08-03

**Authors:** Alan Gill, Joshua Kidd, Fernando Vieira, Kenneth Thompson, Steven Perrin

**Affiliations:** ALS Therapy Development Institute, Cambridge, Massachusetts, United States of America; Hospital Vall d'Hebron, Spain

## Abstract

**Background:**

In any animal model of human disease a positive control therapy that demonstrates efficacy in both the animal model and the human disease can validate the application of that animal model to the discovery of new therapeutics. Such a therapy has recently been reported by Fornai et al. using chronic lithium carbonate treatment and showing therapeutic efficacy in both the high-copy SOD1G93A mouse model of familial amyotrophic lateral sclerosis (ALS), and in human ALS patients.

**Methodology/Principal Findings:**

Seeking to verify this positive control therapy, we tested chronic lithium dosing in a sibling-matched, gender balanced, investigator-blinded trial using the high-copy (average 23 copies) SOD1G93A mouse (n = 27–28/group). Lithium-treated mice received single daily 36.9 mg/kg i.p. injections from 50 days of age through death. This dose delivered 1 mEq/kg (6.94 mg/kg/day lithium ions). Neurological disease severity score and body weight were determined daily during the dosing period. Age at onset of definitive disease and survival duration were recorded. Summary measures from individual body weight changes and neurological score progression, age at disease onset, and age at death were compared using Kaplan-Meier and Cox proportional hazards analysis. Our study did not show lithium efficacy by any measure.

**Conclusions/Significance:**

Rigorous survival study design that includes sibling matching, gender balancing, investigator blinding, and transgene copy number verification for each experimental subject minimized the likelihood of attaining a false positive therapeutic effect in this standard animal model of familial ALS. Results from this study do not support taking lithium carbonate into human clinical trials for ALS.

## Introduction

Amyotrophic Lateral Sclerosis (Lou Gehrig's Disease, ALS [OMIM 105400]) is a degenerative neuromuscular disorder for which there is currently no satisfactory treatment. A proportion of familial cases of ALS have been linked to mutations in the cytoplasmic superoxide dismutase SOD1 (OMIM 147450). Animal models of the familial disease have been used to explore mechanisms of disease onset and progression and to identify potential therapeutics. In any animal model of human disease, a positive control therapy that has been demonstrated to show efficacy in both the animal model and the human disease, provides a valuable tool that can be used to validate the application of that animal model to the discovery of new therapeutics. Such a therapy has recently been reported by Fornai, et al. [1] using chronic lithium carbonate treatment and showing therapeutic efficacy in both the high-copy SOD1G93A mouse model of familial ALS and in human patients. Other investigators [2], [3] have also shown therapeutic efficacy using chronic lithium treatment in this mouse model.

In addition, there is a reasonable basis in the scientific literature for relevant targets and effects of lithium that suggests it could be therapeutic in neurodegenerative diseases [4]–[8]. Compelled by the potential for having a positive control therapy against which to measure other potential therapeutics, and from which to derive genomic targets using gene profiling methods, we conducted the current study. Although lithium's low therapeutic index had the potential to elicit untoward effects during a 4-month survival study, we sought to duplicate exactly the dosing regimen used by Fornai et al. [1] because it showed unequivocal efficacy, and this efficacy was linked to efficacy in ALS patients. We tested chronic lithium dosing in a sibling-matched, gender balanced, investigator-blinded trial using the high-copy SOD1G93A mouse, a standard animal model of familial ALS [9]. Our study did not show efficacy by any measure.

## Results

Chronic intraperitoneal administration of lithium carbonate started at 50 days of age and continued until death. All 28/28 drug group animals, and 27/28 vehicle control group animals, completed the study. One vehicle control animal was sacrificed early at the request of the staff Veterinarian and removed from the study as a non-ALS death.

### Body Weight

Failure to maintain body weight is an indicator of disease onset and progression in the SOD1^G93A^ mouse model of ALS. The average starting body weight (age 50 days) was 19.5±0.57 and 19.8±0.53 g for the vehicle control- and lithium carbonate-treated groups, respectively (females 16.9±0.34 and 17.3±0.30; males 22.0±0.43 and 22.3±0.38).

**Figure 1:**
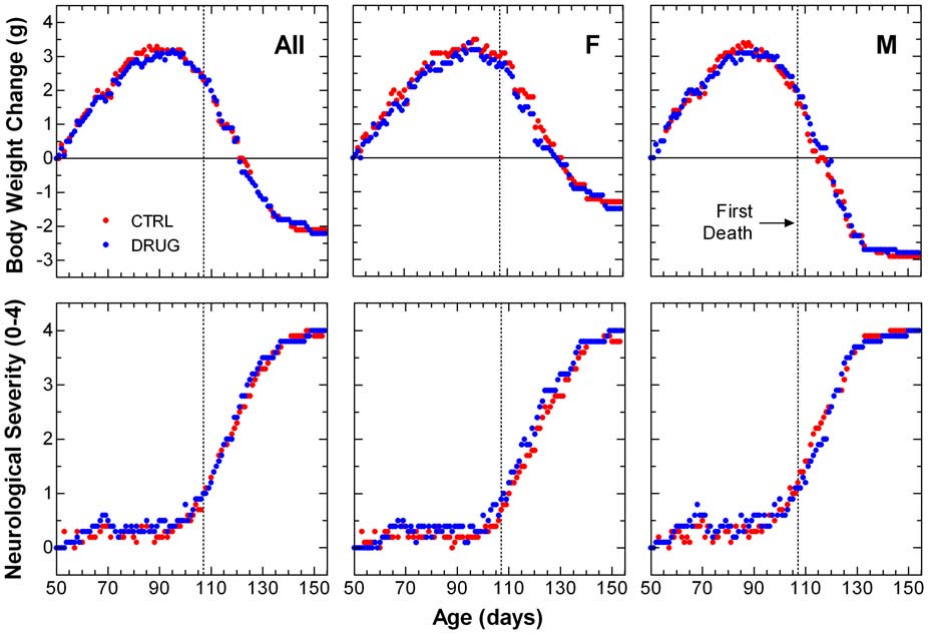
Daily average body weight change and neurological severity score in SOD1^G93A^ mice. Group average change from initial body weight (top panels) and neurological disease severity score (bottom panels) are compared over time from age at study start to age at last death. Left panels show data from all animals (All); middle and right panels show data from females (F) and males (M), respectively. Vehicle control (CTRL) animals received daily 0.2 mL i.p. injections of isotonic saline. Lithium-treated (DRUG) animals received daily 36.9 mg/kg i.p. injections of lithium carbonate dissolved in isotonic saline. Because animals died at different ages, group means were computed after carrying forward last values for each animal until the last animal died. Results of statistical analysis for summary measures taken from these data are given in Table 1.

Treatment group daily average change in body weight over time is plotted through the age at which the last animal died in Figure 1 (top panels). To prevent decomposition of the mean values as animals began to die, last values were carried forward during computation of the means. Drug group animals showed slightly lesser body weight gain (up to 0.5 g) during the ascent to peak body weight. This effect was greater in males than in females.

When examined separately for each animal, peak body weight occurred at different ages. When body weight change over time was fitted for each animal and measures derived from the individual curves, time to peak body weight and time from peak to terminal body weight were similar in both groups (Table 1). The average maximum increase from initial body weight (CTRL 3.5±0.2, DRUG 3.3±0.2 g, p = 0.5000) and age at peak body weight (CTRL 95.1±2.0, DRUG 95.2±1.8 days of age, p = 0.9911) were similar in both groups. Median weight change from peak body weight to terminal body weight (CTRL 2.3±0.2, DRUG 2.2±0.2, p = 0.7520) was also similar in both groups (Student's 2-tailed independent t-test). Based on individual changes in body weight, chronic dosing with lithium carbonate had no effect on disease onset or progression in these studies. However, group average body weights suggested that mice in the lithium-treated group maintained body weight less effectively by as much as 0.5 g.

**Table 1:** Time-to-Event Analysis for Peak Change in Body Weight.^1, 2^

Parameter	Subjects	Trtmnt	N	Median Time	Risk Ratio	Test	Prob>ChiSq
Time to Peak (days)	All	CTRL	27	42	1.04	Log-Rank	0.8203
		DRUG	28	46	0.96	Wilcoxon	0.7747
						Likelihood	0.8900
	Female	CTRL	13	52	0.67	Log-Rank	0.3200
		DRUG	14	47	1.49	Wilcoxon	0.6798
						Likelihood	0.3340
	Male	CTRL	14	38	1.23	Log-Rank	0.5781
		DRUG	14	42	0.81	Wilcoxon	0.7482
						Likelihood	0.5926
Peak to Death (days)	All	CTRL	27	29	0.99	Log-Rank	0.9683
		DRUG	28	31	1.01	Wilcoxon	0.4999
						Likelihood	0.9710
	Female	CTRL	13	28	0.99	Log-Rank	0.9733
		DRUG	14	29	1.01	Wilcoxon	0.7713
						Likelihood	0.9750
	Male	CTRL	14	29	1.22	Log-Rank	0.9490
		DRUG	14	34	0.82	Wilcoxon	0.3935
						Likelihood	0.6975

1 Testing Terms: In Kaplan-Meier analysis the Log-Rank test places more weight on later event times; the Wilcoxon test places more weight on early event times and is the optimum rank test if the error distribution is logistic. Prob>ChiSq lists the probability of obtaining, by chance alone, a Chi-square value greater than the one computed if the time-to-event functions are the same for all groups. In Cox proportional hazards analysis a Risk Ratio of 1.00 occurs when timing is likely identical in the two groups. The Effect Likelihood Test is the likelihood-ratio Chi-square test on the null hypothesis that the parameter estimate for the Treatment covariate is zero (no effect of treatment). Testing term descriptions are taken from the JMP® 7.0.1 Help file.

2 Peak change from initial body weight was detected after spline smoothing (stiffness 100) of each animal's body weight change over time during the period from study start to death.

### Neurological Severity Score

The ALS-TDI neurological scoring system was designed to provide an unbiased assessment of disease onset, progression and severity of paralysis. Treatment group daily average neurological disease severity score over time is plotted through the age at which the last animal died in Figure 1 (bottom panels). To prevent decomposition of the mean values as animals began to die, last values were carried forward during computation of the means. Drug group animals showed a slightly more rapid progression in neurological disease severity score during the early onset phase of the disease. This effect was prolonged into the later stages of disease in females.

**Figure 2:**
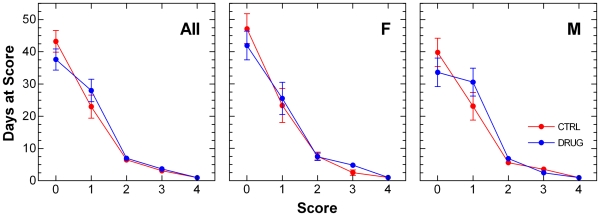
Average number of days spent at each neurological severity score level in SOD1^G93A^ mice. Left panel shows data from all animals (All); middle and right panels show data from females (F) and males (M), respectively. Vehicle control (CTRL) animals received daily 0.2 mL i.p. injections of isotonic saline. Lithium-treated (DRUG) animals received daily 36.9 mg/kg i.p. injections of lithium carbonate dissolved in isotonic saline. Data are treatment group means±s.e.m.. Scores advance in severity from 0 to 4. Results of statistical analysis for these data are given in Table 2.

Days spent at each neurological severity score level were determined for individual mice. These were compared for the vehicle control- and lithium carbonate-treated groups in Figure 2. Drug group animals spent fewer days at the asymptomatic Score0 level and more days at the early symptomatic Score1 level than vehicle control group mice. This effect was greater in males than in females. The difference was statistically significant when analyzed by categorical analysis (Table 2).

**Table 2:** Neurological Severity Score: Categorical Analysis of Days at Score by Treatment.^1, 2^

Subjects	Parameter	Treatment	Score0	Score1	Score2	Score3	Score4
All	Frequency	CTRL	1161	645	175	84	27
		DRUG	1049	804	198	104	28
	Share of Responses	CTRL	0.56	0.31	0.08	0.04	0.01
		DRUG	0.48	0.37	0.09	0.05	0.01
	Prob>ChiSq		0.0012	0.0005	0.4008	0.2259	1.0000
Females	Frequency	CTRL	612	301	100	33	13
		DRUG	586	355	104	68	14
	Share of Responses	CTRL	0.58	0.28	0.09	0.03	0.01
		DRUG	0.52	0.32	0.09	0.06	0.01
	Prob>ChiSq		0.0420	0.2455	0.8033	0.0016	1.0000
Males	Frequency	CTRL	549	344	75	51	14
		DRUG	463	449	94	36	14
	Share of Responses	CTRL	0.53	0.33	0.07	0.05	0.01
		DRUG	0.44	0.43	0.09	0.03	0.01
	Prob>ChiSq		0.0068	0.0002	0.1434	0.1069	1.0000

1 Testing Terms: Frequency is the number of occurrences of that score for the entire treatment group over the duration of the study. Share of Responses is the proportion of total responses by the treatment group that occurred at each Score level. Prob>ChiSq lists the probability of obtaining, by chance alone, a Chi-square value greater than the one computed if the score frequencies are the same for both Treatment groups at group size minus one degrees of freedom.

2 Neurological scores were assigned as described in detail in Materials and Methods. Neurological disease severity increases from 0–4. Score0 is asymptomatic, Score4 is moribund.

### Disease Onset and Survival

**Figure 3:**
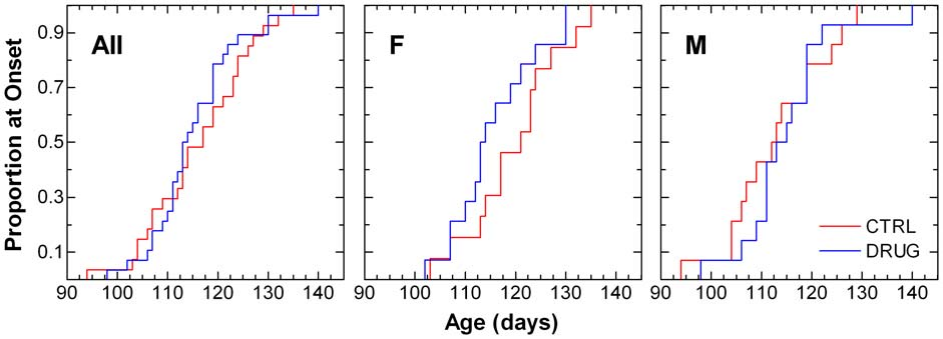
Kaplan-Meier time-to-failure plot for onset of symptomatic neurological disease in SOD1^G93A^ mice. The age at which mice attain a neurological severity score of 2 is taken to be definitive onset of symptomatic disease. Left panel shows data from all animals (All); middle and right panels show data from females (F) and males (M), respectively. Vehicle control (CTRL) animals received daily 0.2 mL i.p. injections of isotonic saline. Lithium-treated (DRUG) animals received daily 36.9 mg/kg i.p. injections of lithium carbonate dissolved in isotonic saline. Results of statistical analysis for these data are given in the upper portion of Table 3.

The proportion of mice that progressed to definitive onset of symptomatic neurological disease over time is shown in Figure 3. Overall, there was no statistically significant difference in onset of disease. Drug group females tended to show earlier (median 7 days) onset. However, this difference was not statistically significant (Table 3).

**Table 3:** Time-to-Event Analysis for Onset of Neurological Symptoms and for Survival Duration.^1, 2^

Parameter	Subjects	Trtmnt	N	Median Time	Risk Ratio	Test	Prob>ChiSq
Onset Age (days)	All	CTRL	27	117	0.90	Log-Rank	0.6345
		DRUG	28	114	1.11	Wilcoxon	0.5785
						Likelihood	0.7170
	Female	CTRL	13	121	0.60	Log-Rank	0.1876
		DRUG	14	114	1.66	Wilcoxon	0.1814
						Likelihood	0.2140
	Male	CTRL	14	113	1.13	Log-Rank	0.7586
		DRUG	14	115	0.89	Wilcoxon	0.6293
						Likelihood	0.7680
Death Age (days)	All	CTRL	27	127	1.01	Log-Rank	0.9304
		DRUG	28	124	0.99	Wilcoxon	0.9397
						Likelihood	0.9730
	Female	CTRL	13	129	0.84	Log-Rank	0.6587
		DRUG	14	124	1.19	Wilcoxon	0.7357
						Likelihood	0.6650
	Male	CTRL	14	126	1.11	Log-Rank	0.7726
		DRUG	14	124	0.90	Wilcoxon	0.7483
						Likelihood	0.7800

1 Testing Terms: In Kaplan-Meier analysis the Log-Rank test places more weight on later event times; the Wilcoxon test places more weight on early event times and is the optimum rank test if the error distribution is logistic. Prob>ChiSq lists the probability of obtaining, by chance alone, a Chi-square value greater than the one computed if the time-to-event functions are the same for all groups. In Cox proportional hazards analysis a Risk Ratio of 1.00 occurs when timing is likely identical in the two groups. The Effect Likelihood Test is the likelihood-ratio Chi-square test on the null hypothesis that the parameter estimate for the Treatment covariate is zero (no effect of treatment). Testing term descriptions are taken from the JMP® 7.0.1 Help file.

2 A neurological score of two in both hind limbs is taken to be the definitive onset of neurological symptoms.

**Figure 4:**
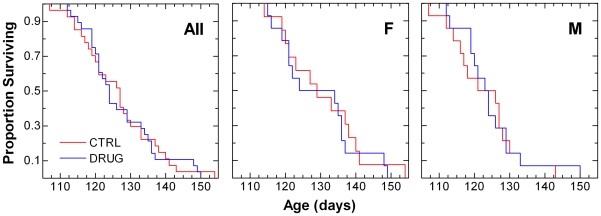
Kaplan-Meier survival plot for age at death in SOD1^G93A^ mice. Left panel shows data from all animals (All); middle and right panels show data from females (F) and males (M), respectively. Vehicle control (CTRL) animals received daily 0.2 mL i.p. injections of isotonic saline. Lithium-treated (DRUG) animals received daily 36.9 mg/kg i.p. injections of lithium carbonate dissolved in isotonic saline. Results of statistical analysis for these data are given in the lower portion of Table 3.

The proportion of mice surviving over time is shown in Figure 4. There was no statistically significant difference in survival proportions over time when comparing vehicle control- with lithium carbonate-treated high-copy SOD1G93A mice (Table 3).

### Supporting Data

Supporting data are available in Dataset S1. This is a 976 KB Microsoft Excel notebook file named “Lithium Dataset S1.xls”. Databases containing all raw and derived data and tables summarizing the data in detail are provided in separate tabs of the Excel notebook. Tabs in the file contain the following elements: DB1 (indexed database), DB2 (list database), BW (mean body weight over time), BWCh (mean body weight change over time), SmBWch (spline 100 smoothed body weight change over time), NSAve (mean neurological severity score over time), BWPeak (peak body weight changes), BWPeakAve (mean peak body weight changes), NeuroAll (days and median age at score by mouse), NeuroAve (mean days and median age at score).

## Discussion

Any animal model of disease capable of predicting therapeutic outcomes in the human patient is a valuable tool that can be applied to the discovery of new therapeutics. Encouraged by the recent report from Fornai et al. [1] of a treatment that has been demonstrated to show efficacy in both the animal model and the human disease, we sought to duplicate the exact therapeutic regimen used in their mouse study. Fornai, et al. used chronic lithium carbonate treatment and showed therapeutic efficacy in both the high-copy SOD1G93A mouse model of familial ALS and in human patients. In our study we tested chronic lithium dosing in an effectively powered, sibling matched, gender balanced, transgene copy number verified, investigator blinded trial using the high-copy SOD1G93A mouse. However, our study did not show efficacy by any measure.

Both mouse studies employed the same mouse strain (B6SJL-TgN(SOD1-G93A)1Gur) and lithium salt, formulation, dose, route, and frequency. We began lithium treatment at 50 days of age. Fornai et al. began treatment at 75 days of age. They also noted that they achieve comparable efficacy even when lithium treatment is started at the onset of motor symptoms. Thus, variations in the time of treatment start would not be expected to affect efficacy results, especially if treatment were given from an earlier age. However, transgene copy number [10] and gender do seem to affect disease onset and survival duration. Any difference in these variables that is not effectively balanced between treatment groups could cause comparative outcome differences that are falsely attributed to the drug treatment regimen.

Fornai et al. performed extensive motor function testing as part of their study. We have avoided such testing because we have noticed that survival appears shortened in studies that perform extensive rotarod and other motor functions tests. Multiple sequential rotarod trials given twice weekly, along with grip strength and stride length tests, involve more frequent handling and induced movement than would occur in our trials. Extensive motor function testing can be considered a kind of environmental enrichment. Such enrichment has been shown by some to shorten lifespan in ALS mice [11]. For example, our vehicle control group's median survival age (127 days of age) was later than the Fornai et al. vehicle control median survival age (111 days). Their vehicle control median survival age even preceded our vehicle control's median age at disease onset (117 days of age). Thus, their experimental conditions appear to promote a shorter lifespan than ours. Median lifespan of the SJL strain high-copy SOD1G93A mouse is 130 days, as originally characterized by Gurney [12], and as maintained by the Jackson Laboratories (personal communication). The ALS-TDI SOD1G93A SJL colony maintained by Genzyme Therapeutics has a median lifespan, calculated over 5000 mice, of 131 days. The early disease onset described in Fornai et al. might be attributable to differences in experimental conditions described above, to copy-number drift in the colony, to differences in handling non-ALS deaths, or to the way that the experimental design dealt with gender or littermate variables.

Our study, that would likely have put steady-state lithium levels at about 1.5 mM in TBW, did not show efficacy by any measure. Not surprisingly given lithium's low therapeutic index, there were small negative effects in some parameters. This raises the question about whether a dosing regimen that attained lower levels would have been more effective. In a review of lithium's potential therapeutic role in neurodegenerative diseases Chuang and Manji [13] point out that much lower lithium levels than those likely attained in our studies may still be effectively neuroprotective and also avoid toxicity. For example, four weeks of lithium dosing to attain steady-state plasma levels of ≈0.35 mM increased bcl-2 levels in frontal cortex and hippocampus in rats, cultured cortical neurons could be protected from glutamate excitotoxicity by 0.1–0.6 mM lithium concentrations, and lithium was significantly protective in the middle cerebral artery occlusion stroke model at 0.5 mEq/kg. Thus, doses of lithium lower than those required to treat symptoms of mania have demonstrated neurotrophic and neuroprotective effects.

A recent publication from the laboratory of Caterina Bendotti [14] reported studies testing lithium carbonate's efficacy in high-copy G93A mice from both the C57BL6/J and 129S2/Sv genetic backgrounds. Because the Fornai group had shown efficacy in only male animals, the Bendotti studies were performed in only female animals. The dosing protocol used was identical to the Fornai studies. Serial motor function tests, terminal motor neuron counts, and markers of autophagy and mitochondrial function were included. No therapeutic effects related to disease onset or survival, or improvements in anatomical or biochemical measures of neuroprotection were shown in the C57BL6/J background mice. Mice from the 129S2/Sv background showed earlier disease onset and shorter survival times. Thus, no therapeutic effects of lithium were demonstrated in female SOD1G93A mice from two strain backgrounds in the Bendotti studies.

Two other recent studies that intended to test treatment combinations, included lithium treatment groups that appeared to show efficacy against disease in the G93A mouse. The first, by Shin et al. [2] used a 0.2% lithium carbonate diet supplement (taken to be about 200 mg/kg/day) beginning at age 56 days and using treatment group sizes of 13 mice. Their study showed about a 10-day delay in onset of symptoms and a similar increase in mean survival. Gender was not clearly identified in the study and the differences reported could result from not effectively gender balancing and litter matching the treatment groups. Our data show that unbalanced and unmatched groups of this size would have a 35% chance of showing a positive effect of treatment by chance alone [9]. Reported beneficial effects on motor neuron counts were not performed using unbiased stereological methods.

A second study by Feng, et al. [3], treated high-copy SOD1 G93A ALS mice with twice daily i.p. lithium chloride (60 mg/kg or 1.41 mEq/kg) injections, starting from 30 days after birth and continuing until death. Presence or absence of the transgene was verified by PCR. Mice were randomly divided into treatment groups, and were matched for littermates with 6 animals (3 males + 3 females) in each group. Behavioral observers were blinded. Like Shin et al., the Feng study showed about a 10 day delay in onset and a similar increase in mean survival. Our data show that even with properly balanced and matched treatment groups, the Feng study would have a 30–40% chance of showing an apparent treatment effect by chance alone, based on the group size, not excluding non-ALS deaths and not excluding low copy mice [9].

Although these studies did not measure lithium levels during treatment, we used pharmacokinetic modeling and simulation, based on reported parameter estimates for lithium in mice [15], to predict plasma lithium levels for the dosing regimens used. Based on these simulations average steady-state plasma Li+ levels would be predicted to be 0.30 mM for our study, 0.97 mM for the Shin study, and 1.68 mM for the Feng study. A level of 0.35 mM has been reported to robustly increase Bcl2 in rat brain (13), levels of 0.1 to 0.2 mM are neuroprotective against glutamate toxicity [16], and 0.4 to 1.25 mM is considered a safe and effective concentration range for human use in psychiatric disorders. Thus, our Fornai, et al.,-based dosing regimen would not likely produce toxic levels of lithium during treatment and was potentially sufficient to produce benefit. In our study, at steady state animals would have spent about 13 h in the concentration range considered to be effective and acceptably safe, each day. The Shin study would likely have kept blood lithium levels within the therapeutic concentration range as well. However, blood levels in the Feng study would likely have spent about 12 hours each day in excess of the maximal safe concentration. Peak levels would be predicted to exceed the maximum safe concentration by about 3.5 fold. Even average daily levels would likely be about 35% above the safe level. Lithium has a narrow therapeutic index. For example, when given at 80 mg/kg IP, about twice the dose we gave in our study, lithium carbonate induced renal insufficiency in Wistar mice [17]. The failure to maintain adequate fluid intake can lead to dangerous concentrations of lithium in the body, but this would likely occur later in disease progression and would be common to all of the studies.

In summary, despite applying the rigorous methods detailed by Scott et al. [9] to our therapeutic trial, we were unable to show efficacy of chronic lithium treatment in the SOD1G93A mouse ALS model. Only low-grade signs of treatment intolerance were evident in our study. Differences in gender balance, transgene copy number, or presence or absence of motor function testing could account for apparent differences between the two studies. Lithium may also bear testing using a dosing regimen that attains lesser, safer, but still potentially neurotrophic, plasma and tissue concentrations.

### Conclusion

Although small changes in lithium exposure could produce toxicity that may offset therapeutic benefit, it is likely that rigorous survival study design that includes sibling matching, gender balancing, investigator blinding, and transgene copy number verification for each experimental subject has minimized the likelihood of attaining a false positive therapeutic effect in this standard animal model of familial ALS. The current study did not identify any therapeutic benefit of chronic lithium treatment with respect to disease onset, progression of neurological symptoms, or survival duration, in the high-copy SOD1G93A mouse model. There was, however, evidence for early onset of low-grade neurological symptoms and signs of less effective body weight maintenance in females that may have reflected untoward effects of lithium treatment.

## Materials and Methods

We used the rigorous survival study methods described in detail by Scott, et al. [9] summarized briefly below, for the current study.

### Animals

All procedures were carried out in accordance with guidelines of the ALSTDI Animal Care and Use Committee and in accordance with the Institute for Laboratory Animal Research (ILAR) Guide for Care and Use of Laboratory Animals [18]. High-copy SOD1G93A transgenic mice bred under contract for ALS TDI by Genzyme Transgenic Corporation (GTC) were used for this study. This mouse colony was derived from the B6SJL-TgN(SOD1G93A)1Gur strain, obtained from The Jackson Laboratory (Bar Harbor, Maine) and originally produced by Gurney et al. [12]. GTC maintains the colony by crossing C57BL/6 sires harboring the transgene with wild-type SJL dams. Copy number is determined by quantitative Southern blot analysis of sires, selecting animals with approximately 23 copies. Animals with a lower copy number are known to have a longer lifespan [10], [12]. To verify presence of the transgene in the F1 progeny, tail biopsies are collected from 14-day-old pups, then genotyped using PCR. Transgenic mice are shipped to ALS TDI at 35–45 days of age. Mice are allowed at least one week to acclimate to ALS TDI's animal facility (a 12-h light/dark cycle) before being assigned to a study. Prior to data analysis, transgene copy number of all animals in the study was checked using PCR analysis of ear-punch samples.

### Experimental Groups

At 50 days of age mice were separated into Drug Treatment (DRUG) and Vehicle Control (CTRL) groups. Prior to any treatment, groups were constituted so as to minimize between-group variability by using the following criteria. Groups were balanced with respect to gender (14 males, 14 females per group) and body weight within gender (mean starting weights were typically within 0.3 g for either gender between groups). In addition, groups were age-matched and littermate-matched. Each male and female in the Drug Treatment group had a corresponding male and female littermate in the Vehicle Control group. The study was observer-blinded.

### Treatments

Lithium carbonate (Sigma-Aldrich, product number 255823-100G, purity 99+% A.C.S. reagent) was dissolved at a concentration of 3.69 mg/mL into isotonic sodium chloride solution. This formulation was given to animals at a volume of 10 mL/kg body weight. The lithium treatment group received daily intraperitoneal 36.9 mg/kg lithium carbonate injections. This dosage was calculated to deliver 6.94 mg/kg/day or 1 mEq/kg/day of lithium ions. Vehicle control group animals received daily 10 mL/kg intraperitoneal isotonic sodium chloride injections.

### Monitoring Disease Progression

#### Neurological Score

Neurological scores for both hind legs were assessed daily for each mouse from 50 days of age. The neurological score employed a scale of 0 to 4 that was developed by observation at ALSTDI. Criteria used to assign each score level were:

Score Criteria

1. 0 Full extension of hind legs away from lateral midline when mouse is suspended by its tail, and mouse can hold this for 2 seconds, suspended 2–3 times.
2. 1 Collapse or partial collapse of leg extension towards lateral midline (weakness) or trembling of hind legs during tail suspension.
3. 2 Toes curl under at least twice during walking of 12 inches, or any part of foot is dragging along cage bottom/table*.
4. 3 Rigid paralysis or minimal joint movement, foot not being used for forward motion.
5. 4 Mouse cannot right itself within 30 seconds from either side.

*If one hind leg is scored as 2, food pellets are left on bedding. If both hind legs are scored as 2, Nutra-Gel® (Bio-Serve #S4798) is provided as food in addition to food pellets on bedding and a long sipper tube is placed on the water bottle.

#### Body Weight

Body weight is a sensitive indicator of any malaise that might result from chronic drug treatment and of motor impairment that occurs during disease progression. Daily body weight measurements were recorded for each animal beginning at 50 days of age.

#### Survival

Date and cause of death were recorded for each mouse. For humane reasons, animals are closely monitored and sacrificed as moribund prior to actual death using criteria for severe moribundity. To determine duration of survival reliably and humanely, the moribund state, defined as the inability of mice to right themselves 30 seconds after being placed on a side (a neurological score of 4) was used. The moribund mice were scored as “dead”, and were euthanized using carbon dioxide.

### Statistical Analysis

To visualize overall changes in body weight and neurological severity score over time, group mean body weight change and mean neurological severity score were plotted over time through the age at which the last death occurred in either treatment group (Figure 1). Because the animals die at different ages and the timing of their ascent to peak body weight and descent to minimum body weight differs markedly from animal to animal, individual body weight change over time is also shown in Figure S1.

In order to minimize the number of statistical comparisons and to retain statistical validity in the analysis of correlated serial measurements on the same subject over time, we determined relevant summary measures as recommended by Matthews, et al. [19] and performed statistical analyses on these summary measures. Summary measures derived from body weight change over time included time to attain peak body weight and time from peak body weight to death. These summary measures were detected from spline-fitted changes in body weight over time for each individual animal. These time-to-event measures were then analyzed using Kaplan-Meier survival fit analysis with the Log-Rank and Wilcoxon tests for statistical significance. Cox proportional hazards analysis was also performed to determine hazard ratios and test for statistical significance of their differences using the Effect Likelihood Chi Square test.

A summary measure derived from daily ordinal neurological severity scores counted days at score for each animal at each score level (Figure 2). These data were analyzed using categorical analysis where Chi Square tests were used to determine whether any differences in score frequencies were statistically significant. Degrees of freedom were based on the number of animals in each group rather than total number of scores (Table 2).

Time to onset of definitive disease and survival time were also analyzed using the Kaplan-Meier and Cox proportional hazard methods (Figures 3, 4, Table 3). Statistical analyses were performed using JMP® 7.0.1, SAS Institute, Inc., SAS Campus Drive, Cary, NC 27513, USA. Cox proportional hazard fitting, using litter as a frailty term, was performed using Stata®/IC 10.1, 4905 Lakeway Drive, College Station, TX 77845, USA. P-values less than 0.05 were taken to be statistically significant.

Supporting data for all measured and derived parameters are provided in Dataset S1.

### Mouse Study Laboratory Information Management System (LIMS)

All research data related to the animal studies were managed via LIMS. Any individual mouse, with its unique ID, could be traced back to its lineage, body weight or neurological score on any given day, and any other observations noted during its lifetime. Similarly, tissues from each mouse could also be tracked for future analysis.

## Supporting Information

Figure S1 Smoothed body weight change over time for each individual animal. Left panels show data from all animals (All); middle and right panels show data from females (F) and males (M), respectively. Top three panels show vehicle control animals (red); bottom three panels show lithium-treated animals (blue).
(0.72 MB TIF)

Dataset S1 This is a 976 KB Microsoft Excel notebook file named “Lithium Dataset S1.xls”. Databases containing all raw and derived data and tables summarizing the data in detail are provided in separate tabs of the Excel notebook. Tabs in the file contain the following elements: DB1 (indexed database), DB2 (list database), BW (mean body weight over time), BWCh (mean body weight change over time), SmBWch (spline 100 smoothed body weight change over time), NSAve (mean neurological severity score over time), BWPeak (peak body weight changes), BWPeakAve (mean peak body weight changes), NeuroAll (days and median age at score by mouse), NeuroAve (mean days and median age at score).
(1.08 MB XLS)
